# The Retention of Nitrogenous Waste Products in the Blood

**Published:** 1916-03

**Authors:** Charles G. Stockton, John L. Butsch

**Affiliations:** Prof. of Medicine in the University of Buffalo, Attending Physician Buffalo General Hospital; Assistant Prof. Pharmacology, University of Buffalo


// BUFFALO MEDICAL JOURNAL

Yearly Volume 71 MARCH, 1916 No. 8

### ORIGINAL ARTICLES

The right is reserved to decline papers not dealing with practical medical and surgical subjects, and such as might offend or fail to interest readers. Contributors are solely responsible for opinions, methods of expression and revision of proof.

### The Retention of Nitrogenous Waste Products in the Blood

By CHARLES G. STOCKTON, M. D.,

Prof. of Medicine in the University of Buffalo,
Attending Physician Buffalo General Hospital.

JOHN L. BUTSCH, M. D.,

Assistant Prof. Pharmacology, University of Buffalo.

Early in the last century Prevost and Dumas were the first to demonstrate that in nephrectomized animals, the urea in the blood was increased. Their methods were crude and not much of a quantitative study could be made. The fact, however, was of great importance, and from that time until the present much attention has been paid to the study of the waste nitrogen in the circulating blood. Not until the middle of the last century were very accurate quantitative studies made. With these Picard was the first to be able to show that the blood urea varies with the proteid intake. Tileston and Comfort were able to show later that other factors caused a retention of the urea. Especially the severe anemias, intestinal obstruction, syphilis, lolar pneumonia and chronic plumbism, the coma of diabetes mellitus, and acute yellow atrophy of the liver.

In our studies of the chemistry of the nitrogenous wastes in the blood, the technic for urea was the urease method of Marshall, described in the Journal of Biological Chemistry. For uric acid and total non-proteid nitrogen we used the method of Folin and Denis described in the same journal. Along with these studies we have been making estimation upon the hydrogen ion concentration in the blood by the method of Rowntree and Levin, Archives of Internal Medicine, and of the Co2 tension in the alveolar air by the Fredrica method. We found the normal values for urea to be 12 to 14 mg. of urea nitrogen. Uric acid 1-2 mg., and total non-proteid nitrogen 23 to 25 mg., each estimated for 100 cc. of blood.

The amount of protein ingesta has a distinct bearing on the nitrogenous retention in the blood. We cite a case. A man, age 72, retired since the age of 45. At this age he had what he called a nervous breakdown, for which he took extensive treatment, both in this country and abroad. During the years from the age of 45 to the present time he has given most careful attention to his absorptive and eliminative processes. On a most carefully selected diet, well directed exercises and baths, properly alkalinized with suitable waters, this man at the age of 72 has a systolic blood pressure of 120-diastolic 90, with a remarkable urine for a man of his age. On account of the illness of his wife and the war he did not go abroad, but presented himself to us for study. His blood was a surprise; having a uric acid content of 1.8 mg. and a urea of 7.8 mg., total non-proteid nitrogen of 19.2, chlorids 68 mg., blood sugar .10, hydrogen ion concentration of his blood 7.8. This man when he presented himself was taking about 40 gms. of proteid, 160 of fat, 100 of carbohydrate, and about 2000 calories. For study he was placed on a considerably higher dietary, consisting of proteid 90, fat 190, carbohydrate 250, and about 3500 calories. On this diet his blood showed the following: uric acid 2.4, urea 14.2, total non-proteid nitrogen 29.6. His blood sugar remained the same, his hydrogen ion concertration rose slightly. His urinary acidity, which was 25 acidity per cent. at the beginning of his study, quickly rose to 85 acidity per cent, after being on the increased diet for three days. His urinary indican, which was 13 mg. for 24 hours at the beginning, quickly rose to 42 mg. for 24 hours. This study shows, as we have noticed in numerous other cases, that the nitrogenous wastes are considerably modified by diet. Not only are the nitrogenous wastes piled up in the blood serum, but many other metabolic disturbances were noticed. His urinary ammonia rose, as did the hydrogen ion concentration. Before the increased dietary was given his nitrogen balance was only slightly positive. On the increased diet he showed a large amount of nitrogen retention. This case shows an increasing retention of the waste nitrogen with increased feeding, but we can reverse the conditions and show records of cases which came to the hospital with markedly increased retention which, upon proper dietary, fell promptly to within normal limits. Others it required from four to six weeks to reduce their nitrogen percentage in the blood. A few cases fail to respond to treatment at all. It is especially those who have neglected themselves too long. So long that their urea retention was above 60 mg. When the urea nitrogen is much above 60 mg. the prognosis begins to be grave. It is as necessary for these patients to remain on a low proteid diet after they have been relieved of their retention as it is for a diabetic to remain on a low carbohydrate diet after he has been relieved of his hyperglycaemia.

The causes of nitrogen waste retention are many. Most often it is due to some form of nephritis, but many other pathological conditions may increase it. We have it in severe anemias, leucaemias, acute infections, especially in some stages of pneumonia. We have found it in what was seemingly a pure cardiac decomposition. In pyloric stenosis, intestinal stenosis, esophageal stenosis, and in cases which have persistent vomiting from any cause we find an increase in the nitrogen wastes in the blood. Some of these pathological conditions have associated with them increased catabolism, which if accompanied by much intermediate acid formation gives rise to slight acidosis, which in turn decreases renal function, which may be demonstrated if not morphologically at least physiologically. In this way both factors are at play. Increased total non-proteid nitrogen formation and a decrease in its elimination.

Most cases of acute nephritis show retention. If one encounters high values in acute kidney lesions the chances for recovery are greater than if one encounters it in the chronic cases, Values as high as 106 mg. urea nitrogen have been found which promptly fell as the treatment of the nephritis progressed. This is especially true of young patients.

Mild cases of nephritis usually show a retention of between 30 and 40 mg. of urea nitrogen. It is in these cases that prophylaxis should begin. When a patient shows these values, he should be under careful dietetic direction, together with regular exercise and elimination.

Advanced cases of chronic nephritis show values from 60 to 140 mg. of urea nitrogen. Cases showing values above 60 mg. must be given the most careful treatment. When the retention of urea nitrogen reaches 90 to 100 mg. the prognosis is grave. Exidus occurs usually in less than a month. We have, however, held such patients for three months under the most rigorous "regieme." Occasionally we find a severe chronic nephritis with a retention of 90 to 100 mg., which recedes to a mild type with intensive treatment. We have such cases now who are living in comfort so far as symptoms go. They are symptomatically free from disease so long as they remain within the bounds of their prescribed restrictions. That is: low diet, alkalies, graded exercise, both mental and physical. Several such cases who, three years ago, gave every sign of a rapid approaching end, are still well and happy. Not, however, very active nor gormandizers .

In prostatic hypertrophy we may or may not have a retention, depending upon how much the kidney is involved. If there is a retention of urine in the bladder there is usually a nitrogen retention in the blood, which will subside if the kidneys are not involved. It is in these cases that the greatest discrepancies between the phenolsulphonphtalein tests and the retention tests occur. Patients having a phtalein test as low as 7% in 2 hours, have failed to show a retention, which were operated in spite of their low pthalein and made uneventful recoveries. The cases of prostatic hypertrophy in our series which have shown a high retention of nitrogen products in the blood, have been thoroughly eliminated before operation, and we have yet to see a case of postoperative acidosis following this condition if the retention was reduced and kept down by proper treatment both before and after the operation.

In a number of our cardi-nephritic cases we have had opportunities at the same time we studied the blood, to study the pleural and abdominal fluids. We find as did other observers, that they agree closely with the blood in their content of nitrogenous wastes. The spinal fluid was studied in cases where a spinal puncture was done for diagnostic purposes. It too, agrees closely with the blood. We found, however, that in the majority of our cases which gave a positive Wasserman reaction, there was always more or less of a retention.

In hypopitutiaryism we found that the values were lower than normal, the same was true of hypothyroidism, but the reverse was true with hyperthyroidism. We studied the effect of a right lobectomy on one of our cases. The case was a young married woman age 25 years, showed a retention of 35 mg. before operation. Just after her operation the value went up to 42 mg. Two months after her operation it was again down to normal.

---

**The Incest Epic of the Freudians.** Geo. Mitchell Parker of N. Y., Med. Rec. Jan. 1, 1916, cites parents "who with a contented spirit shared their knowledge of cancer and Montessori, of salvarsan and Gary schools . . . . alcohol and twi-light sleep." The very natural and frank preference of their 4-year old son for the mother, is interpreted as incestuous. The article is rather long but well illustrates the possible disadvantages of lay instruction in medical matters, and especially the tendency to interpret childish impulses in sexual terms. Allusion may also be made here, to several editorial articles, condemning the playing of female parts by college students as a manifestation of sexual depravity, and as liable to cause homosexual stimulation in the audience.

